# Increasing uptake of colon cancer screening in a medically underserved population with the addition of blood-based testing

**DOI:** 10.1186/s12885-021-08678-8

**Published:** 2021-08-28

**Authors:** Stephanie Ioannou, Kyle Sutherland, Daniel A. Sussman, Amar R. Deshpande

**Affiliations:** University of Miami Miller School of Medicine, 1600 NW 10th Ave, Miami, FL 33136 USA

**Keywords:** Colorectal cancer, Screening, Non-invasive testing, Medically underserved populations, Health fair

## Abstract

**Background:**

Adherence to colorectal cancer screening in the United States is suboptimal, particularly in medically underserved populations due to significant barriers to care. Unique accessible, low-cost, and non-invasive screening tests for this population could greatly benefit current rates. In this article, we assess patient preference and the impact of offering a blood-based test on screening rates in a cost-free health fair setting from April 2017 to April 2019.

**Methods:**

Participants who met colorectal cancer screening eligibility criteria set forth by the United States Preventive Services Task Force were recommended to attend the colon cancer screening station. Those participants who elected to attend were offered various, accepted screening methods, and if they declined, were offered alternative blood-based testing. Screening rates, test outcomes, and the rate of follow up completion of colonoscopy were measured and compared with historic screening outcomes.

**Results:**

Of 1401 participants who were recommended to attend, 640 (45.7%) participants were evaluated at the colon cancer screening station, of whom 460 were eligible for testing. Amongst these, none selected colonoscopy, 30 (6.5%) selected fecal immunochemical testing, and 430 (93.5%) selected blood-based testing. Only 2 participants returned the fecal immunochemical tests. In the blood test cohort, 88 were positive and 20 received a follow up colonoscopy.

**Conclusions:**

Based on this assessment, blood-based testing is an effective method to increase screening rates in medically underserved populations, though efforts to further improve access to follow up colonoscopy are necessary.

**Supplementary Information:**

The online version contains supplementary material available at 10.1186/s12885-021-08678-8.

## Background

Colorectal cancer (CRC) is the second leading cause of cancer mortality nationally [1]. Routine screening for CRC, such as with colonoscopy and fecal immunochemical testing (FIT), at regular intervals leads to earlier CRC detection, lower CRC incidence and mortality, and overall cost savings compared to no screening [2–4]. However, there are significant barriers to screening within medically underserved populations (MUP), due in part to cost, accessibility, and acceptability of screening tests. Behavioral Risk Factor Surveillance System (BRFSS) survey data identified low CRC screening rates among participants without healthcare coverage (37%) compared to those with health insurance (69%), as well as lower rates in participants who lack an identifiable healthcare provider (31%) compared to those with a regular provider (69%) [5]. Low income and lack of insurance lead to later CRC detection, worse outcomes, and increased mortality from CRC [6].

To date, there are many well-established CRC screening modalities, which can be divided into invasive (capsule endoscopy, sigmoidoscopy, or colonoscopy) or non-invasive testing options (computed tomographic colonography and stool-based testing), and can be further categorized into those detecting both polyps and cancer versus cancers alone [7]. In the United States, the most utilized method for CRC screening is colonoscopy, but it is not necessarily an ideal screening tool in MUP given its cost, invasiveness, risk, lack of convenience and accessibility, and patient perception [8, 9]. To that end, the other modalities remain important alternative screening options. In fact, modeling studies clearly demonstrate that all the screening methods have benefit over time, and that adherence to testing, regardless of modality, is a key driver for successful screening [10]. Many people are not up-to-date with CRC screening despite the available testing, with overall screening rates under 70% in the United States in 2018 [11]. Some characteristics of a MUP, such as low income, lack of insurance or being underinsured, and lack of a primary care provider (PCP), make screening an even bigger challenge for this group. This is particularly the case for access to colonoscopy.

FIT has desirable characteristics for the MUP including cost, availability, and efficacy. It has been used frequently for CRC screening in this population, and programmatic FIT has been shown to be effective [12]. However, return rates are consistently low, around 10%, in MUP compared to the general population [13]. For MUP without access to a primary care provider who may use health fairs as their principal source of health care, low FIT return rates limit the feasibility of this option.

The Mitchell Wolfson Sr. Department of Community Service (DOCS) is a medical student-run, organization at the University of Miami Miller School of Medicine that provides preventive, primary, and subspecialty care to thousands of MUP in South Florida through various organized activities, including free comprehensive screening health fairs. This patient population is almost exclusively uninsured or underinsured, and a large percentage are not eligible for state or federal programs that facilitate primary care. The county safety net health system.

affords care for some, but this is mostly in the emergent or urgent settings rather than for elective issues like cancer screening. Therefore, the lone modality of colorectal cancer screening at these fairs has been fecal occult blood testing for many years, initially with guaiac-based and, then, with immune-based testing (FIT), given its affordability. However, we had noted consistently low return and positivity rates of FIT at our health fairs, in line with known national averages [13]. In the year prior to the data collection presented here, when FIT was the primary CRC screening option offered, 414 participants received FIT but only 52 returned them (12.6%) with 0 being positive (0%).

Blood-based testing presents an opportunity to overcome some, but not all, of the barriers that currently limit screening, particularly in MUP, including ease of use and educational barriers to test use and return. The FDA-approved Septin9 DNA blood test (Epi proColon®) is indicated for screening of participants unwilling or unable to undergo the other recommended tests, and is therefore appropriate for use in this study design. This test determines the methylation status of the SEPT9 gene, a member of the Septin family of proteins that bind GTP and act to modulate vesicle trafficking, apoptosis, cytoskeletal remodeling, and cytokinesis [14]. Hypermethylation of the SEPT9 gene in CRC tissue is associated with colorectal carcinogenesis [15] and methylated SEPT9 DNA (mSEPT9) shed from the tumor site is measurable in peripheral blood, providing a blood-based means to test for CRC [16, 17]. Several studies have compared mSEPT9 and FIT testing, and test sensitivity has been found to be comparable across all stages, though at lower mSEPT9 specificity. In one prospective multi-center study, mSEPT9 sensitivity was 73% at a specificity of 80%, compared to 68% at 97% for FIT [18].

Given the potential for a blood-based test to improve screening rates for MUP, we developed a program to assess the impact of adding mSEPT9 testing to our CRC screening station at the DOCS health fairs. Here we report on CRC screening uptake with both mSEPT9 and FIT in MUP and secondarily the performance characteristics of the blood-based test in this resource-limited and ethnically diverse environment.

## Methods

The CRC screening assessment received approval from the Institutional Review Board at the University of Miami. All participants recruited at the DOCS health fairs provided consent upon check in at the fair. A retrospective chart review was performed of all participants attending DOCS health fairs from April 2017 to April 2019. In 2016, DOCS data collection was transitioned from paper charts to an electronic medical record system using REDCap, a secure metadata-driven Electronic Data Capture (EDC) software and workflow methodology for designing clinical and translational research databases while ensuring HIPAA compliance [19]. All participants over age 50 years (the recommended age to start screening in average risk individuals at that time) attending DOCS health fairs in South Florida during the pre-determined two-year period were asked to visit the CRC screening station during their time at the fair.

At the CRC screening station, those who were at average risk of colorectal cancer (defined as age 50–75 without rectal bleeding or previous history of colorectal polyps, CRC, inflammatory bowel disease, familial cancer syndromes, or family history of CRC) and not up-to-date with CRC screening (no colonoscopy within the last 10 years, sigmoidoscopy within the last 5 years, or FIT within the last 1 year) were educated on CRC screening by station volunteers using a prewritten script that also explained their options for screening (see Appendix 1). Translators were used if English was not the patient’s primary language of preference.

A screening questionnaire was administered at the CRC station, and a prewritten script was used with the goal of providing an objective, unbiased approach to CRC screening options. Participants were offered the option of assistance in getting a screening colonoscopy within the county safety net hospital system; however, given lack of access to the healthcare system for the majority of participants, a script was used to explain that colonoscopy could not be guaranteed. Participants were also offered the option of a take-home FIT screening kit at the fair in the patient’s primary language to be completed as per kit instructions and mailed back to DOCS in the provided prepaid addressed return envelope. In line with the intended use of the blood test, participants were offered and declined both colonoscopy and FIT before being offered blood-based mSEPT9 testing. Screening modalities were offered in a stepwise fashion in accordance with current guidelines for CRC screening, with gold standard being colonoscopy and FIT having increased sensitivity, affordability, and familiarity.

On acceptance of mSEPT9 testing, a blood sample was collected by venipuncture-trained student volunteers and processed on-site per manufacturer instructions for use. Plasma was stored and shipped in labeled 5-mL screwcap and flat-bottom transport tubes at − 15 °C to − 25 °C to Molecular Pathology Laboratory Network, Inc. (MPLN) (Maryville, TN) for testing. Results were reported online in MPLN’s LIS Blue laboratory management system 1–2 weeks after the health fair. FIT kits were instructed to be returned by mail to the University of Miami and were processed at the University of Miami laboratory.

All participants who elected for the mSEPT9 blood draw or mail-returned a FIT kit had their results mailed to them in their primary language, including an explanation of appropriate follow up recommendations, within 2–4 weeks of sample collection or return of FIT kit. Participants who screened positive for either test were contacted by telephone 2, 4, and 6 weeks after receiving their results by trained medical student patient navigators to assist in facilitating follow-up colonoscopies; participants were followed until time of colonoscopy completion or for up to 9 months after sample collection, whichever came first. Screening rates, test rate positivity, and follow-up colonoscopy rates for both FIT and mSEPT9 were analyzed through the use of descriptive statistics.

## Results

A total of 2513 people attended 19 health fairs. Of all health fair attendants, 1401 (55.8%) participants were over 50 years old and directed on initial intake to the CRC screening station. Of those directed to the CRC screening station, 640 participants attended the CRC screening station (45.7%). At that station, 159 participants were excluded for being greater than average risk (42) or being up-to-date with screening (117). Of the 481 remaining eligible participants, 21 participants had incomplete medical records largely due to missing data points. Therefore, 460 participants were included for retrospective analysis. Of these, 50% were uninsured and 47% had not seen a PCP within the preceding year. No participant chose to pursue colonoscopy as the initial screening test. Thirty participants (6.5%) chose to pursue FIT, and 430 (93.5%) participants elected for mSEPT9 testing (Table 1).

**Table 1:** Population demographics and results

Age	
Mean – yr	62
Distribution- no. (%)	
50–59	199 (43)
60–69	177 (38)
70–79	63 (14)
> 80	21 (5)
Sex– no. (%)	
Male	179 (39)
Female	281 (61)
Race/Ethnicity- no. (%)	
White (Non-Hispanic)	152 (33)
White (Hispanic)	147 (32)
Black (Afr American)	32 (7)
Black (Caribbean/Haitian)	101 (22)
Black (Hispanic)	6 (1)
Other	14 (3)
Declined to Answer	8 (2)
Primary Language Spoken in Household- no. (%)	
English	236 (51)
Spanish	126 (27)
Haitian Creole	81 (18)
Other	8 (2)
Declined to Answer	9 (2)
Health Insurance Enrollment- no. (%)	
Uninsured	229 (50)
Insured (Private, Medicaid/Medicare, or County Assistance Program)	211 (46)
Declined to Answer	20 (4)
Seen Primary Care Physician in the Last Year- no. (%)	
Yes	226 (49)
No	215 (47)
Declined to Answer	19 (4)
Preferred Test– no. (%)	
FIT	30 (7)
Septin-9	430 (93)
Result, of mSEPT9 Testing– no. (% of mSEPT9)	
Positive	88 (20)
Negative	342 (80)
FIT Kits Returned– no. (% of FIT Kits Distributed)	2 (7)

Two of the 30 participants who chose FIT returned the test (6.7%) and neither was positive (0%). Of the 430 participants who had blood-based testing, 88 (20.5%) screened positive. In the nine-month follow-up period, 20 (22.7%) of the 88 participants who screened positive received diagnostic colonoscopies, 48 (54.5%) were pending colonoscopies due to financial constraints, and 20 (22.7%) were considered lost to follow up after three failed attempts to contact. In those who received diagnostic colonoscopies to date, no high-risk lesions (adenomas > 1 cm, villous features, high-grade dysplasia, serrated lesions, or cancers) have been identified.

## Discussion

The mSEPT9 blood test had similar characteristics as previously reported in studies where participants had access to care; however, the number of participants requiring diagnostic colonoscopy for positive mSEPT9 is worthy of consideration. Despite participant agreement to participate in accessible screening modalities, barriers to further care pose additional complications. The lack of access to follow-up services for those with positive mSEPT9 screening has proven to be a challenge, particularly in light of the high test positivity rate, as has the ability to routinely remain in contact with this population, primarily due to inconsistent addresses and phone access. However, incorporation of blood-based testing in resource-poor settings may facilitate identification of individuals who would benefit most from colonoscopy and can be greatly beneficial when paired with a patient navigation program that can facilitate coordination of needed care. Many of the participants who received follow up colonoscopy after positive mSEPT9 screen were able to do so because of the assistance of patient navigation to facilitate access to financial assistance within the county safety net hospital system.

By providing mSEPT9 as an alternative method of CRC screening in MUP in South Florida, there was increased uptake of testing despite the underlying socio-economic challenges that this population faces. Lack of adequate uptake of subsequent diagnostic colonoscopy in those participants testing positive is concerning and points to the need for improved navigation coupled with the need for easier access to colonoscopy. In those who received diagnostic colonoscopy, lack of high-risk lesions is difficult to interpret until more are completed: a challenge with our transient and demographically-varied MUP. While statistically non-inferior to FIT with respect to sensitivity, specificity is ~ 80% compared to ~ 97% for FIT [20, 21]. Future work will be aimed at longer follow up of participants, larger populations, and quantification of patient acceptability. In addition, an ongoing post approval study is focused on measuring repeat testing uptake over time for participants with negative test results.

One limitation of the study was that testing was provided free of charge to all participants, which would remove a cost barrier for participants. In this regard, the results may not be generalizable to the population. However, this is a typical of provision of services in a health care setting, and by way of comparison, FIT testing was also offered at no cost. On this basis, the impact on test uptake remains valid. As noted above, a second limitation was the lack of easy availability of screening colonoscopy. Recognizing this challenge, we simply reported the absence of colonoscopy screening and make no comparison claims with this method. While the Affordable Care Act (ACA) mandates full coverage for screening colonoscopy for those insured, this does not fully remove barriers to screening as health fair participants often have no coverage. Therefore, while our observations may not be generalizable to the broader population, they are relevant to MUPS as they have many of the same barriers to care in common.

From a public health perspective, the increased uptake of screening is notable, though importantly, we recognize the concern that comes from screening participants who are unable to access a diagnostic colonoscopy following a positive screening test. To some degree, inability to access colonoscopy is a complication that may deter future use; however, given the variability among MUP, it is difficult to determine ease of colonoscopy access at the time of screening. Furthermore, the lack of access to follow-up colonoscopy is a challenge in common for all non-invasive screening tests. The cost of testing represents a second public health challenge. Testing by FIT remains the least costly approach to screening, and there is and increased cost impact of blood-based testing, like other non-invasive methods such as stool DNA [22]. However, blood-based testing has been shown to be a cost-effective approach for screening, given an observed increase in adherence to testing [23].

Modeling studies have shown that adherence to screening is a key driver in the success of screening programs [10]. Our assessment of blood-based testing has clearly demonstrated that this alternative modality has the potential to improve adherence to screening in MUP, overcoming the barriers associated with the other screening methods. Combining blood-based screening with navigation for participants with positive results may be an appropriate model to improve MUP CRC screening rates.

## Conclusions

The availability of mSEPT9 screening in MUP unwilling or unable to complete FIT or colonoscopy led to a marked increase in screening uptake when compared to years prior, where FIT was the only screening modality offered at the health fairs. For eligible participants, the rate of testing increased from 12.6% completing testing with FIT the previous year to 93.5% with the blood test. Although navigation to colonoscopy was offered, it was not a comparator in the study as we had no prior data. In our health-fair setting, most participants are uninsured or underinsured, and thus experience great difficulty accessing colonoscopy.

As noted in literature on self-reported barriers to invasive screening modalities, financial constraints to screening are rarely the only barrier to uptake. Other barriers include logistical challenges (e.g., lack of transportation, time off of work), lack of provider referral, and concern with bowel preparation [24]. While increased education is vital in patient understanding of the importance of screening, addressing and overcoming these additional barriers has proven to be more challenging, leading to increased desirability of non-invasive testing.

The introduction of a novel approach to CRC screening (blood-based testing) when compared to FIT has proven to be well-received by our MUP, likely because of convenience and ease of testing. In this study, we demonstrate that tests performed same day at health fairs are likely to have higher uptake than those tests that are less accessible to these populations. In the health fair setting, participants have been accustomed to providing blood samples for other screening tests, and it is likely that the execution of blood-based CRC testing is more in line with a participants’ expectations of medical care than is sample acquisition for stool-based testing. It is probable that the inconvenience posed by completing and returning a FIT also plays a role in patient preference.

These findings are in line with previous studies on blood-based testing. In a cross-sectional survey of 100 participants, blood-based testing was ranked as a first or second choice of screening in 91% when compared with colonoscopy, sigmoidoscopy, or stool testing. Participants preferred mSEPT9 over FIT and/or colonoscopy due to convenience (60%), low cost (47%), low level of discomfort (30%), lack of required bowel preparation (28%), and more frequent screening intervals (18%) [25]. Further data will be needed to better understand patient choice in the health fair setting when selecting among CRC screening tests.

## Supplementary Information

**Additional file 1.**

## Data Availability

All data generated or analyzed during this study are included in this published article [and its supplementary information files].

## Abbreviations

BRFSS: Behavioral Risk Factor Surveillance System
CRC: Colorectal cancer
DOCS: Mitchell Wolfson Sr. Department of Community Service
EDC: Electronic Data Capture
FIT: Fecal immunochemical testing
MPLN: Molecular Pathology Laboratory Network, Inc.
mSEPT9: Methylated SEPT9 DNA
MUP: Medically underserved populations
PCP: Primary care provider
