# Supplementary material for: Increasing uptake of colon cancer screening in a medically underserved population with the addition of blood-based testing
Source: BMC Cancer. 2021 Aug 28;21:966. doi: 10.1186/s12885-021-08678-8 (PMC8401245; doi:10.1186/s12885-021-08678-8)
Supplement: Supplementary file 1 — Additional file 1. [file 12885_2021_8678_MOESM1_ESM.docx]

**AT REGISTRATION: send all people 50-75 to CRC station (next to glucose/lipids/Hep C/HIV). The questions below will be asked there.**

**CRC SCREENING STATION (AT VENI)**

1. Are you at least 50 years old?

🞏 YES 🞏 NO **STOP, NO NEED FOR SCREENING**

1. Have you ever been told that you have colorectal cancer?

🞏 YES **GO TO HIGHLIGHTED PARAGRAPH BELOW** 🞏 NO/I DO NOT KNOW

1. Do you have a family history of colorectal cancer?

🞏 YES **GO TO HIGHLIGHTED PARAGRAPH BELOW** 🞏 NO/I DO NOT KNOW

1. Have you seen blood in your stool in the past 6 months?

🞏 YES **GO TO HIGHLIGHTED PARAGRAPH BELOW** 🞏 NO/I DO NOT KNOW

***: Thank you for your time today. Based on the information you provided, you are not eligible for our screening fair, but we strongly suggest you seek medical care for further evaluation. Our colleagues can help make a referral for you.

**If all NO’s:** Colorectal cancer is cancer of the colon or rectum – two parts of the large intestine. There are two main types of screening tests for this type of cancer: stool tests and endoscopies. I am going to ask you about these specific tests.

1. A stool test like fecal occult blood test (gFOBT) or fecal immunochemical testing (FIT) is a test done at home to find whether the stool contains blood. Have you had a stool test for blood in your stool?

🞏 YES 🞏 NO/I DO NOT KNOW

If yes, was it in the last 12 months?

🞏 YES **GO TO HIGHLIGHTED PARAGRAPH BELOW** 🞏 NO/I DO NOT KNOW

1. Endoscopies like sigmoidoscopy and colonoscopy are exams in which a tube is inserted in the rectum to view the colon for signs of cancer or other health problems. You may have been sedated for this procedure. Have you ever had a sigmoidoscopy or colonoscopy?

🞏 YES (select one) 🞏 SIGMOIDOSCOPY 🞏 COLONOSCOPY 🞏 NO/I DO NOT KNOW

If sigmoidoscopy, was it in the last 5 years?

🞏 YES **GO TO HIGHLIGHTED PARAGRAPH BELOW** 🞏 NO/I DO NOT KNOW

If colonoscopy, was it in the last 10 years?

🞏 YES **GO TO HIGHLIGHTED PARAGRAPH BELOW** 🞏 NO/I DO NOT KNOW

**If all NO’s:** Proceed to CRC screening station.

***: Thank you for your time today. If screening is up-to-date, the patient does not need to proceed to the CRC screening stations. If based on Questions 5 and 6 there were findings on prior screening that did not get appropriate follow-up, please mark as HIGH RISK and flag for Check Out to address. If there was another screening test done (e.g ColoGuard which is FIT+DNA), also flag for Check Out to address so appropriate time interval can be determined.

*Perhaps SCOPE can man the table, which should be at the Venipuncture Station.*

Colorectal cancer is the second most common cancer to cause death in the United States. By screening, we can prevent cancer from developing or identify cancers at an early stage so they can be cured. There are a few ways to screen, some of which we’ve already described:

FIT is a stool test looking for microscopic blood in the stool, which can be an early sign of cancer. Unlike older versions of stool tests for this purpose, FIT only detects human blood so food and medicine restrictions are not needed with the test. This test is free to you at our health fairs. You will receive a box with instructions on how to collect your stool at home and mail it back to us. You need to return this to us within 30 days. Please keep in mind that if the stool test detects blood, then we will need to help you schedule a colonoscopy to discover why there was blood in your stool.

Colonoscopy is a test that allows a doctor to see the inner lining of your colon and rectum, two parts of the large intestine. With colonoscopy, a doctor uses a thin tube with a camera to look in the large intestine, checking for cancer and offering the ability to remove pre-cancerous growths. We cannot provide you this test for free but can help arrange it for you; depending on your financial status it could cost several hundred to a few thousand dollars without insurance.

Will you do either of these tests for colorectal cancer screening?

🞏 YES 🞏 NO **GO TO HIGHLIGHTED PARAGRAPH BELOW**

If yes, which one? 🞏 FIT (stool test) 🞏 COLONOSCOPY

If FIT, then give him/her FIT cards.

If colonoscopy then make a notation for this to be facilitated at Check-Out (to give referral).

IF NO: Because you’ve declined these tests, you are eligible for an FDA-approved and commercially available blood test known as Epi proColon® that is indicated for colorectal cancer screening. We are able to provide this test for you as part of this health fair since you are not up-to-date with screening and have turned down alternative methods of screening like stool-based tests and colonoscopy. About 1 in 5 people have an abnormal blood-test result that would require a colonoscopy which could cost several hundred to a few thousand dollars without insurance, but we will help you navigate this process. Are you interested?

🞏 YES 🞏 NO

If no, why?

If yes then send them to Venipuncture to get blood drawn.

**PLEASE REMEMBER THAT THE PATIENTS CAN DECLINE AT ANY TIME AND CAN THEN GET USUAL CARE (FIT OR COLONOSCOPY).**
